# Temporal Capsule Feature Network for Eye-Tracking Emotion Recognition

**DOI:** 10.3390/brainsci15121343

**Published:** 2025-12-18

**Authors:** Qingfeng Gu, Jiannan Chi, Cong Zhang, Boxiang Cao, Jiahui Liu, Yu Wang

**Affiliations:** 1 Beijing Engineering Research Center of Industrial Spectrum Imaging, School of Automation and Electrical Engineering, University of Science and Technology Beijing, Beijing 100083, Chinaustbljh@ustb.edu.cn (J.L.);; 2 Shunde Innovation School, University of Science and Technology Beijing, Foshan 528399, China

**Keywords:** eye tracking, emotion recognition, temporal feature network, capsule network, MLP classification

## Abstract

Eye Tracking (ET) parameters, as physiological signals, are widely applied in emotion recognition and show promising performance. However, emotion recognition relying on ET parameters still faces several challenges: (1) insufficient extraction of temporal dynamic information from the ET parameters; (2) a lack of sophisticated features with strong emotional specificity, which restricts the model’s robustness and individual generalization capability. To address these issues, we propose a novel Temporal Capsule Feature Network (TCFN) for ET parameter-based emotion recognition. The network incorporates a Window Feature Module to extract Eye Movement temporal dynamic information and a specialized Capsule Network Module to mine complementary and collaborative relationships among features. The MLP Classification Module realizes feature-to-category conversion, and a Dual-Loss Mechanism is integrated to optimize overall performance. Experimental results demonstrate the superiority of the proposed model: the average accuracy reaches 83.27% for Arousal and 89.94% for Valence (three-class tasks) on the eSEE-d dataset, and the accuracy rate of four-category across-session emotion recognition is 63.85% on the SEED-IV dataset.

## 1. Introduction

Emotion is a complex physiological phenomenon, and accurately identifying an individual’s emotional state holds significant value for human–computer interaction and cognitive science. Emotional expression relies not only on explicit features like language and facial expressions [1] but also on being conveyed through non-invasive physiological signals, such as eye movements (e.g., blinking, pupil changes) [2,3]. Compared with explicit features such as language and facial expressions, ET features are subconscious behaviors that are more difficult to control, and thus can reflect more authentic emotional changes. Due to their advantages—being “non-invasive, easy to collect, and directly associated with emotion”—ET signals have become an ideal research subject for affective computing. ET signals are mainly composed of fixation, saccade, and pupil information. Fixation count and fixation duration can directly reflect the attention state of subjects as well as their level of focus on the observed targets [4]; saccade is the most common type of ET signal, and different saccade speeds and durations indicate varying degrees of attention toward different targets [5]. While an individual is in different emotional states, their eye features, such as fixation duration, gaze point distribution, saccade amplitude, and pupil diameter variation, exhibit systematic differences [6]. For instance, a pleasant mood tends to encourage an individual to fixate longer on positive stimulus areas, while fear may trigger pupil dilation and high-frequency saccades [7]. The synergistic effects among ET features and the inherent correlations between different emotions and features both demonstrate that ET-based emotion recognition has strong feasibility and scientific validity. Although eye movement features demonstrate advantages in affective computing, several major issues still need to be fully addressed to promote ET-based emotion recognition.

The first issue lies in the lack of “specificity” in the association between ET features and emotion. Currently, most research primarily focuses on multimodal fusion of EEG and ET data [8,9,10], with ET features often used only as a supplement to other signals. However, the association between ET features and emotion is not one-to-one: a single ET feature may be triggered by multiple factors, and a single emotional state may manifest through diverse ET patterns. Therefore, ET features contain profound correlation information, whereas existing studies calculate each ET feature independently in the model without considering the spatial relationships among these features. For instance, the EmotionMeter study showed that the standalone classification accuracy of ET features was only 67%, indicating significant room for improvement [11]. Although the MFFNN proposed by Bao achieved 87.32% accuracy on the SEED-IV dataset, it still failed to adequately explore the deep association between ET signals and emotion, neglecting the synergistic effects of EEG and ET signals across different stages of emotional expression [12]. There is an urgent need for a model capable of mining spatial information among different ET features, and the ability to extract such spatial feature information cannot be achieved by existing CNN and MLP methods.

The second issue is the insufficient extraction of temporal dynamic information from ET parameters. Current ET analysis research often relies on “full-duration statistical features” (such as average fixation duration) for emotion association analysis, failing to adequately mine the dynamic temporal information of ET features (such as the rate of change in pupil diameter) and local region features [13]. This results in the extracted ET features lacking sufficient “temporal representational capacity” for emotion, leading to smaller datasets and increased susceptibility to interference, thereby reducing the accuracy of affective computing [14,15].

The third issue concerns the constraint of individual differences on the generalization capability of ET features. Individual differences constitute a core bottleneck limiting the generalization capability of ET affective computing, pervading both the establishment of ET baselines and the emotional response process. This variation is often ignored by generalized ET models, as research by Lazar found that baseline pupil diameter changes in an “initial decrease followed by stability” pattern with age, with significant differences between childhood and old age [16]. Cui confirmed in their 2024 study that the baseline pupil diameter of diabetic patients (3.8 ± 0.5) in mm is significantly greater than that of healthy individuals (3.5 ± 0.2) mm. This pathological difference may lead to misclassification [17], highlighting the limitations of universal training paradigms.

Aiming to solve these existing issues, this paper proposes a network model incorporating feature extraction and classification, specifically designed for emotion analysis using ET parameters. The network addresses the identified problems through a Temporal Feature Extraction Module, a Capsule Module, and an MLP Module. While the temporal window is widely used in multimodal problems, and the Capsule Network was originally applied in image recognition to capture spatial dependencies between features and has also been applied to speech information to capture feature spatial relationships [18], the Capsule Network can also be applied to the field of action recognition. By leveraging vectorized salient features to represent objects and various action features, it can more effectively determine their final labels, which demonstrates the excellent performance of the Capsule Network in capturing spatial information [19]. Therefore, TCFN combines the temporal window with the Capsule Network. The former leverages mature application experience from multimodal tasks to segment continuous ET data (such as fixation duration and saccade amplitude) into time-correlated feature fragments, effectively capturing the dynamic patterns of emotion over time. The latter leverages the capability of the Capsule Network to model the hierarchical spatial relationships between features, accurately mining the inherent mapping between ET features (e.g., pupil dilation degree, blink frequency) and different emotional states (e.g., pleasure, anxiety). This addresses the problem of weak correlation between ET features and emotions and excavates deeper-level ET information. In addition, TCFN adopts a dual-loss optimization strategy that places greater emphasis on emotion recognition tasks with high classification difficulty, which alleviates the degradation of generalization ability caused by individual differences to a certain extent.

The main advantages of the proposed network are prominent:

(1) The window module not only smooths out the noise interference in the original data but also retains the dynamic eye movement features in the time series, effectively solving the problem of insufficient emotional specificity of features throughout the full-duration period.
(2) The capsule network module encodes the spatial structure among different ET features to explore the deep connections among them. Capsule networks have significantly enhanced the specific representation and generalization ability of eye movement features for emotional states.
(3) The dual-loss optimization mechanism ensures that the model learns the correct classification boundaries.

The structure of this paper is as follows. Section 2 introduces the related work on emotion recognition based on ET features. Section 3 introduces the task definition and method flow of this paper. Afterwards, Section 4 introduces the components of the TCFN. Furthermore, Section 5 provides an overview of the experimental process, results, and analysis. Section 6 presents a discussion on the results and limitations of this work. Finally, Section 7 presents the conclusions of this paper and proposes future work on this subject.

## 2. Related Work

ET research is widely applied across various fields, including applied psychology, neuropsychology, and medicine [20,21,22,23,24]. In recent years, emotion recognition has become a popular research field. However, most studies focus on improving the accuracy of their emotion analysis by adding multimodality, without delving into the deep information in eye movement data. Therefore, although ET has become one of the most widely used techniques in sentiment analysis, using ET parameters alone for sentiment analysis remains a relatively new analytical method. This chapter reviews the current research status of emotion recognition using ET parameters, with a focus on introducing ET features and network models.

### 2.1. Eye Tracking Features for Emotion Analysis

In the process of emotion recognition using ET parameters, researchers first focused on discovering eye movement behavior patterns under specific emotional states. Aracena utilized pupil information for valence recognition [25]. Baharom’s study found that video stimulation of “joy” causes the pupil to show a significant dilation trend over time, and the peak fixation duration was around 7000 milliseconds [26]. These quantified findings provided a phenomenological basis for subsequently inferring emotional states from ET features. For emotion classification using ET features, early research primarily relied on statistical features for model prediction. Jia first explored emotion classification in a Virtual Reality (VR) scenario using pupil position as the sole feature metric [27]. Additionally, Tarnowski and Skaramagkas selected 18 and 29 statistical features, respectively (such as standard deviation of pupil diameter and mean number of fixations), to classify emotions. These methods extracted statistical features from the overall data, and their core limitation was their neglect of temporal dynamic changes in ET parameters across different time points [14,28]. Furthermore, while Li transformed ET signals into images using FFT and other such signal processing methods [29], their approaches failed to deeply explore the temporal correlations among ET features.

### 2.2. Eye Tracking Network Models for Emotion Analysis

In the early stages of affective analysis using ET features, machine learning was the preferred implementation method due to its simple model architectures. In addition to SVM, KNN, and Random Forest, methods like Logistic Regression (LR) and Naive Bayes (NB) were widely explored. However, these methods commonly suffered from issues of feature singularity and poor generalization ability. For example, the prediction model for the emotion Arousal, based on gaze features, as proposed by Skaramagkas, achieved 72% Arousal binary classification accuracy on a small-scale dataset by extracting 29 statistical features (including standard deviation of pupil diameter and mean number of fixations) combined with an SVM classifier; however, this model neglected the temporal correlation between features [14,15]. Li proposed the Deep Gradient Convolutional Neural Network (DGCNN), which converted ET signals into images using FFT and other such signal processing methods, achieving 87% test accuracy on a dataset of four emotional states (tension, calmness, happiness, and sadness) from 16 participants [29]. Tarnowski extracted 18 eye movement-related features (including fixation, saccade, and pupil diameter) and classified the High Arousal/Low Valence, the Low Arousal/Medium Valence, and the High Arousal/High Valence. Three classifiers—SVM, LDA, and KNN—were tested. They used the “Leave-One-Subject-Out” method to assess classification quality, obtaining a maximum classification accuracy of 80%, and the experiment showed that the classification accuracies for High Arousal and Low Arousal were the highest [28]. The limitation of these studies is that they only employed machine learning models and trained them exclusively on their own in-house datasets, without demonstrating their results on public datasets.

Based on the above studies, we summarize the research methods and features employed in different ET-only emotion classification studies in Table 1.

According to the statistical results, for emotion classification using ET-only signals, most studies adopt machine learning models and only apply statistical features instead of temporal features in multimodal tasks. Therefore, to better evaluate the performance of TCFN in this paper, we include models based on ET-only signals in the cognitive load domain as references. Safari proposed a deep hybrid model for workload-level classification based on pre-trained Convolutional Neural Network (CNN) and Long Short-Term Memory (LSTM) models [30]. Elmadjian proposed a lightweight Temporal Convolutional Network (TCN) classifier for ET pattern recognition [31]. In the subsequent experimental comparisons of this paper, LSTM and TCN are employed as baseline models for comparative experiments.

## 3. Proposed Methodology Overview

### 3.1. Task Definition and Fundamental Overview

#### 3.1.1. Task Definition

In this study, the data samples in the dataset are defined as **X**, denoted as $\left\{X\in R^{N\times D}\right\}$, where N denotes the total number of samples and D represents the feature dimension of the data. The target set Y is defined as $Y=\left\{Y_{i}\right\},Y_{i}\in \left\{0,1,\ldots ,C\right\}$, where Y represents the set of target emotion categories, and C is the number of emotion classes. The main objective of this paper is to perform feature extraction and predict the emotion category Y from the input samples **X** using the TCFN.

#### 3.1.2. Emotion Classification

Emotion is a complex psychological, cognitive, and behavioral state related to feelings and thoughts. The classification of emotions has long been a major subject of exploration for researchers. Following the theory of natural selection, emotions also possess an evolutionary history and are largely similar across different cultures. Ekman proposed six basic emotions: anger, fear, sadness, joy, disgust, and surprise [32]. Furthermore, more complex emotions can be formed by combining these six basic emotions. Due to the discontinuity inherent in the discrete emotion model and the ambiguity of boundary emotions, the dimensional emotion model has also been proposed alongside Ekman’s model. Dimensional emotion models typically include two-dimensional and three-dimensional variants. The mainstream two-dimensional emotion model adopts the Valence–Arousal model proposed by Russell [33]. Russell argued that each discrete emotion should not vary independently of others; rather than being discrete states, emotions are highly systematic and linked together through positive or negative correlations. In this model, Valence describes the degree to which an event is positive or negative, and Arousal describes a person’s level of interest or physiological activation regarding the event. Compared to the discrete emotion model, the advantage of this representation is that emotions are not treated as a single category but as coordinates within a system. It offers a more sophisticated way to classify emotions than Ekman’s discrete system, which merely divides them into positive or negative categories. Through Valence and Arousal, Russell’s model can represent the subtle changes between different emotions and allows for a clearer expression of hard-to-define emotions, leading to a more accurate and comprehensive understanding of emotion. Therefore, this paper adopts Russell’s Valence–Arousal dimensional model for emotion classification.

#### 3.1.3. Eye Tracking Feature Definition and Extraction

The model utilizes the I-VT (Velocity–Threshold Identification) algorithm and statistical metrics to extract ET features. Considering the temporal continuity inherent in ET signals, we employ a 5 s sliding window with a 1s step size to compute ET features.

(1) $$X_{\tau }=[x_{t(\tau -1)+1},x_{t(\tau -1)+2},\ldots ,x_{t(\tau -1)+w}]\in R^{N\times D}$$

Formula (1) defines the data corresponding to the τ time window within the dataset, denoted as $X_{\tau }$. This is represented as a contiguous subset of data from X, spanning from sampling point t(τ−1)+1 to t(τ−1)+W. The feature formula for saccade information is denoted as

(2) $$\nu_{i,\tau }=\frac{\sqrt{{\left(x_{t(\tau -1)+i+1}^{\left(1\right)}-x_{t(\tau -1)+i}^{\left(1\right)}\right)}^{2}+{\left(x_{t(\tau -1)+i+1}^{\left(2\right)}-x_{t(\tau -1)+i}^{\left(2\right)}\right)}^{2}}}{\Delta t}$$

The unit of the $\nu_{i,\tau }$ is deg/s. The eye movement speed is reflected from the *i*-th sampling point to the (*i* + 1)-th sampling point. The speed threshold $V_{0}$ is introduced, and the velocity sequence $\left\{\nu_{1,\tau },\nu_{2,\tau },\ldots ,\nu_{n,\tau }\right\}$ is classified within $X_{\tau }$ to obtain the event set $E_{\tau }$ within each window. In this paper, $V_{0}$ is consistent with the method proposed by Skaramagkas [15], which is set to 45 deg/s, and the minimum fixation duration is set to 55 ms. For different scanning speeds, event $E_{\tau }$ in the event set is divided into scanning category and fixation category, as shown in Formula (3).

(3) $$E(i,\tau )=\left\{\begin{array}{ll} fixation, & V_{i,\tau }\leq V_{0}\\ saccade, & V_{i,\tau }>V_{0} \end{array}\right.,$$

For each window $X_{\tau }$, statistics are computed based on the original signal $X_{\tau }$ and IVT event $E_{\tau }$, and finally, the feature vector of the window is formed $F_{\tau }$. The features are, respectively, classified into two categories: (1) event features based on the IVT event $E_{\tau }$; (2) timing characteristics based on the original signal $X_{\tau }$. Ultimately, integrating all the statistics can yield all the features of the *τ*-th window $F_{\tau }$. Arranging all the window features in chronological order can result in a temporal feature matrix covering the entire collection period, $F={\left[F_{1},F_{2},\ldots ,F_{k}\right]}^{T}\in R^{K\times L}$, where *K* represents the total number of time windows, and *L* represents the feature dimension.

### 3.2. Overview of the Proposed Method

**Figure 1:**
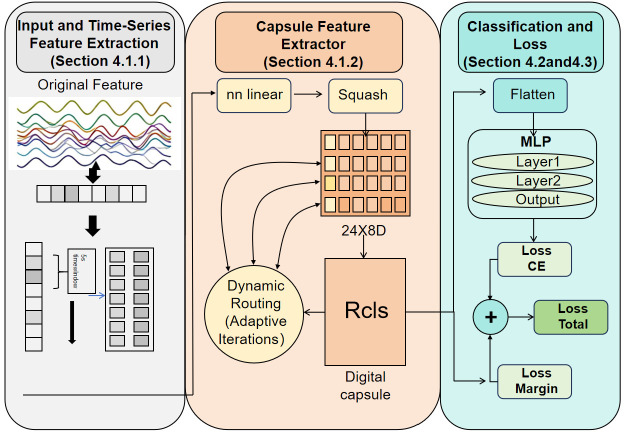
Structure Diagram of the TCFN. This network has three main components: the window function module, the capsule network module, and the MLP module.

The TCFN is designed to generate a more compact and higher-dimensional ET feature representation by leveraging both temporal correlations and inter-feature correlations. The overall architecture of the model is depicted in Figure 1. To achieve this objective, the proposed approach comprises three main components: the Window Feature Module, the Capsule Network Module, and the MLP Module. The Window Feature Module focuses on extracting dynamic information on individual ET features in the temporal domain. The Capsule Network Module concentrates on mining the complementary and synergistic information between different ET features. Finally, MLP can be used for emotion classification. This approach significantly enhances the performance of ET features for emotion classification. The following subsections detail the Temporal Capsule Feature Network.

## 4. Method

### 4.1. Temporal Capsule Feature Network Architecture

#### 4.1.1. Window Feature Module

The window feature module is a component of the TCFN model proposed in this paper, dedicated to extracting temporal features during the data preprocessing stage. Its specific method involves rolling a fixed-size window across the time series of features to calculate the mean value, thereby extracting temporal features. These extracted temporal features are then concatenated with statistical features. This process is strictly conducted under the conditions of subject independence and experiment independence. The window feature module does not alter the overall sample size; instead, it calculates each statistical feature separately and concatenates the resulting features with the original statistical features, only doubling the feature dimension. For example, given a sample with 13-dimensional features and a sample size of 1000 (denoted as the dimension [1000, 13]), the dimension of the sample after window feature processing becomes [1000, 26].

Experimental results demonstrate that the window feature module smooths noise interference in the original features through the rolling average method while preserving the temporal characteristics of the data. Meanwhile, by retaining the temporal sequence, this module improves the resolution of ET features, effectively enhances the ability of the subsequent capsule network module to extract temporal correlation information, and ultimately boosts the overall classification accuracy of the model.

#### 4.1.2. Capsule Network Module

The Capsule Network was first proposed by Hinton to compensate for the deficiency of CNNs in capturing spatial features of data. To this end, Hinton proposed the Capsule Network, which replaces scalar neurons with vector neurons and encodes features through the directional information of vectors, fundamentally addressing the limitations of CNNs. It has demonstrated superior recognition accuracy and anti-interference capability compared to CNNs on the MNIST dataset [34]. In addition, Aykut proposed the Random Capsule Classification Network by combining the Capsule Network with Random Forest, which also exhibited excellent performance [35].

**Figure 2:**
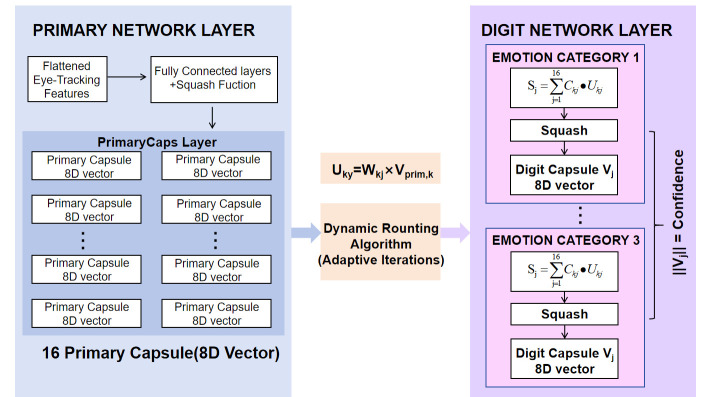
Capsule Network Module Diagram. The digital capsule layer with dimensions [3 × 8] was obtained through three iterations from 16 8D primary capsules.

To explore the spatial feature relationships among different ET features, this paper adopts the Capsule Network as a novel approach for ET emotion classification. The Capsule Network can better learn and reflect the complex spatial structures in the network input data, thereby mining deep-seated ET features. Its specific structure is illustrated in Figure 2.

The Capsule Network Module adopts a two-level structure: the Primary Capsule Layer (PrimaryCaps) and the Digit Capsule Layer (DigitCaps). The core function of the PrimaryCaps layer is to transform the flattened input feature vector **X** into a set of primary capsules containing local structural information through fully connected layers. This achieves the critical conversion from “scalar features” to “vector capsules.” While the original PrimaryCaps layer employs convolutional kernels sliding across an image grid to extract local spatial features, ET data lacks a spatial grid structure. Therefore, the PrimaryCaps layer in this model is modified specifically for this structure: convolution is replaced by fully connected layers, temporal encapsulation is used to preserve temporal information, and the squash function is utilized to compress the vector length (preserving direction information and normalizing the length to the [0, 1] interval). This process can be formally expressed by Formulas (4) and (5).

(4) $$S_{prim}=X\cdot W_{prim}+b_{prim}$$

$W_{\mathrm{prim}}\in R^{F\times (m\times n)}$, where *F* represents the number of features, *m* represents the number of capsules, and *n* represents the capsule dimension. In this paper, *t* is set to 1, *m* is set to 16, and *n* is set to 8. $b_{prim}$ represents the bias term.

(5) $$V_{\mathrm{prim}}=squash(S_{prim})$$

where $V_{\mathrm{prim}}$ represents a set containing K multi-dimensional vectors. In the primary capsule structure proposed in this paper, a total of 16 primary capsules are configured. These 16 primary capsules correspond to 16 combinations of local features derived from ET data (e.g., “Pupil Changes + Saccade Velocity,” “Fixation Positions + Blink Frequency,” etc.). The choice of an 8D vector ensures the richness of the feature representation while avoiding increased computational overhead caused by excessively high dimensions.

The Digit Capsule Layer takes the dynamic routing algorithm as its core, updates routing weights in each iteration, and aggregates 16 primary capsules to form class capsules (with the dimension of class capsules being [3 × 8]). The feature mapping of the Digit Capsule Layer is shown in Formula (6).

(6) $$U_{\mathrm{ky}}=linear(V_{prim,k},W_{kj})$$

where $U_{\mathrm{ky}}$ represents the prediction vector mapping the *k*-th primary capsule to category *y*, and $W_{\mathrm{kj}}$ is the exclusive weight matrix from the *k*-th primary capsule to the *j*-th category capsule. The principle of dynamic routing lies in optimizing “routing weights” through multiple iterations, enabling class capsules to aggregate information from primary capsules that is highly relevant to the corresponding class. This process thereby encodes the collaborative logic among different eye-tracking features. The iterative calculation of dynamic routing is as follows:

(7) $$C_{\mathrm{kj}}=soft\max(B_{kj})$$

(8) $$B_{\mathrm{kj}}=B_{\mathrm{kj}}+dot(U_{ky},V_{j})$$

(9) $$S_{j}=sum(C_{kj}\cdot U_{kj})$$

(10) $$V_{j}=squash(S_{j})$$

where $C_{\mathrm{kj}}$ represents the coupling coefficient (routing weight), and $B_{\mathrm{kj}}$ represents the initial routing logits. $V_{j}$ represents the current class capsule vector. After normalizing the routing logits using Formula (7), the weights are updated by incorporating the predictive vector and the current capsule vector using Formula (8). Formulas (9) and (10) are used to aggregate the predictive vectors based on the optimized coupling coefficients, thereby generating the raw capsule vector $S_{j}$ for class *j*. Subsequently, the Squash function is applied to compress this vector, resulting in the exclusive class capsule vector $V_{j}$. The final output dimension of the Digit Capsule Layer is a high-dimensional vector containing both category and dimensional information, with a shape of [3 × 8]. Each capsule vector corresponds to an emotion category, and its length directly reflects the confidence of the category prediction, providing an unambiguous class representation for subsequent classification. Furthermore, the coupling coefficient $C_{\mathrm{kj}}$ allows for the quantification of the contribution of each primary capsule (i.e., Eye Tracking feature combination) to the class capsule. For instance, if **C**_23_ = 0.8 (the weight of the 2nd primary capsule to the 3rd class capsule), it indicates that the corresponding feature combination (“Pupil Diameter + Fixation Duration”) of the 2nd primary capsule is a key feature for distinguishing the 3rd emotion class, thereby offering an interpretable basis for the model’s decision.

In the classic Capsule Network, the number of iterations for dynamic routing is fixed. However, different categories vary in complexity, and certain categories are more difficult to distinguish. To address the above limitations, this paper proposes an adaptive routing iteration mechanism that provides differentiated iteration counts for different categories. More iterations are allocated to features with high complexity and high classification difficulty. The calculation formula for adaptive iteration is expressed as Formula (11).

(11) $$R_{j}=R_{base}+\alpha \cdot Var\left\|V_{j}\right\|$$

where $R_{j}$ denotes the final number of iterations, $R_{base}=3$ denotes the base number of iterations, α=1 denotes the adjustment coefficient, and $Var\left\|V_{j}\right\|$ is the variance of the capsule vector length of the j-th category.

### 4.2. MLP Classification Network Module

In the network structure of this paper, after the Capsule Network obtains the class capsules, an MLP classifier is introduced for classification with cross-entropy loss. This is because Margin Loss only relies on the length of capsule vectors to determine class confidence, which leads to unsatisfactory performance in ambiguous boundary classification tasks such as emotion classification. Additionally, it wastes the feature spatial structure information contained in the class capsules. By flattening the three 8D class capsules (consistent with the number of emotion classes C = 3) into a 24D vector and feeding it to the MLP, the model can leverage the MLP’s non-linear capabilities to fully mine the complex interactions among all 24 feature dimensions, thereby constructing a more refined decision boundary. Moreover, the Cross-Entropy Loss adopted by the MLP provides smooth and robust gradient signals, effectively overcoming the issue of insufficient gradients in the “flat regions” of Margin Loss, which significantly improves the network’s optimization stability and convergence speed.

The MLP model adopts a three-layer fully connected architecture (Input Layer → Hidden Layer 1 → Hidden Layer 2 → Output Layer), incorporating multiple optimization modules to balance fitting ability and generalization ability. The model’s hidden layers consist of two levels of neurons: the first hidden layer contains 128 neurons, and the second hidden layer contains 256 neurons. Each layer utilizes the LeakyReLU activation function to mitigate the vanishing gradient problem. Furthermore, a BatchNorm1d layer is introduced to accelerate model convergence and enhance training stability, and a Dropout rate of 0.5 is used to enhance the model’s generalization ability. The output layer contains 3 neurons, corresponding to the three target classes in the dataset. It directly outputs the logits (pre-Softmax values), which are then combined with the Cross-Entropy Loss function to compute the loss for the classification task. The specific network computation steps are detailed in Algorithm 1.
**Algorithm 1:** MLP Classification Network for Capsule FeaturesInput:         
$V_{j}$: Output of capsule network (shape: [batch_size, j, 8] Output:         Logits: Classification logits (shape: [batch_size, j])       1 Step 1: Feature Flattening       
$2\;\;\;\;\;\mathrm{Flat}\text{\_}\mathrm{feat}\;\leftarrow \;\mathrm{Reshape}\;(V_{j}$, [batch_size, j × 8])       3 Step 2: First Hidden Layer (Dimensionality Elevation)       
$4\;\;\;\;\;x_{1}$ ← linear (Flat_feat, in_dim = j × 8, out_dim = 128)       
$5\;\;\;\;\;x_{1}$
$\;\leftarrow \;\mathrm{BatchNorm}1d\;(x_{1}$, momentum = 0.05)//Stabilize training distribution       
$6\;\;\;\;\;x_{1}$
$\;\leftarrow \;\mathrm{LeakyReLU}\;(x_{1}$, negative_slope = 0.2)//Alleviate gradient vanishing       
$7\;\;\;\;\;x_{1}$
$\;\leftarrow \;\mathrm{Dropout}\;(x_{1}$, rate = 0.5)//Enhance generalization against noise       8 Step 3: Second Hidden Layer (Dimensionality Reduction)       
$9\;\;\;\;\;x_{2}$
$\;\leftarrow \;\mathrm{linear}\;(x_{1}$, in_dim = 128, out_dim = 256)       
$10\;\;\;\;x_{2}$
$\;\leftarrow \;\mathrm{BatchNorm}1d\;(x_{2}$, momentum = 0.05)       
$11\;\;\;\;x_{2}$
$\;\leftarrow \;\mathrm{LeakyReLU}\;(x_{2}$, negative_slope = 0.2)       
$12\;\;\;\;x_{2}$
$\;\leftarrow \;\mathrm{Dropout}\;(x_{2}$, rate = 0.5)       13 Step 4:Output Layer (Classification Mapping)       
14    Logits ← linear (x_2_, in_dim = 256, out_dim = j)//No Softmax for direct cross entropy computation       15 Return Logits

### 4.3. Dual-Loss Optimization Module

To allow the model to simultaneously learn semantically meaningful capsule feature representations and precise classification decision boundaries, this paper designs a Dual-Loss Optimization Mechanism (DLOM). This mechanism organically integrates Margin Loss [34] with Weighted Cross-Entropy Loss [36], which, respectively, regulate the feature extraction quality of the Capsule Network and the output accuracy of the MLP classifier.

The Margin Loss is specifically designed for the class capsules output by the Digit Capsule Layer of the Capsule Network. The core idea of this loss is to strengthen the physical meaning of the capsule vector’s length through dual-threshold constraints: ensuring that the length of the true class capsule approaches 1 (high confidence) and the length of the non-true class capsule approaches 0 (low confidence). This mechanism guarantees better discrimination among the capsule features. The output of the digital capsule layer obtained from Section 4.1 is $V_{j}\in R^{C\times m\times n}$, *C* represents the number of categories, *m* represents the batch size of the capsules, and *n* represents the dimension of the capsules. Then the length of the *j*-th capsule vector in the *i*-th sample is defined as follows:

(12) $$\mathrm{ca}p\_length_{i,j}=\left\|V_{j}\right\|=\sqrt{\sum_{d=1}^{n}V_{j,m,d}^{2}}$$

To improve class discriminability, a dual-threshold constraint mechanism is introduced for the margin loss: for true classes (encoded by one-hot encoding, denoted as $y_{onehot}(i,j)$, where *i* and *j* are index variables belonging to {0,1}), the capsule length is constrained to be no less than 0.9; for non-true classes, the capsule length is restricted to no more than 0.1, and the interference of negative-class loss is alleviated via the weight parameter β = 0.5. The margin loss calculation Formula (13) is shown as follows.

(13) $$L_{\mathrm{margin}}=\frac{1}{m}\sum_{i=1}^{m}\left[\sum_{j=1}^{n}y_{onehot}(i,j)\cdot \max{\left(0,m-cap\_length_{i,j}\right)}^{2}+\beta \sum_{j=1}^{C}(1-y_{onehot}(i,j))\cdot \max{\left(0,cap\_length_{i,j}-m\right)}^{2}\right]$$

Regarding the classification score vector $S\in R^{m\times C}$ output by the MLP classifier, the Weighted Cross-Entropy Loss introduces the category weight vector *w* = [*w*_1_, *w*_2_,..., *w*_C_] to alleviate the impact of imbalanced sample distribution and is specifically expressed as Formula (14).

(14) $$L_{\mathrm{ce}}=-\frac{1}{m}\sum_{i=1}^{m}\sum_{j=i}^{C}\omega_{j}\cdot y_{onehot}(i,j)\cdot \log(soft\max(S_{i,j}))$$

where $soft\max(S_{i,j})=\frac{\exp(S_{i,j})}{\sum_{k=1}^{C}\exp(S_{i,k})}$ is the normalized category probability, $\omega_{j}$ is positively correlated with the classification difficulty of class-j samples, and its calculation method is adjusted in real time based on the misclassification rate of each class in the validation set during each training round, with the specific formula shown in Formula (15).

(15) $$\omega_{j}=\frac{{\mathrm{error}}_{j}}{\frac{1}{c}\sum_{k=1}^{c}error_{j}}$$

where ${\mathrm{error}}_{j}$ denotes the misclassification rate of class-j samples, and C denotes the total number of sample classes. The final overall loss function is Formula (16).

(16) $$\mathrm{Loss}=L_{\mathrm{margin}}+\lambda L_{ce}$$

## 5. Experimental Results and Analysis

### 5.1. Experimental Procedure

#### 5.1.1. Experimental Design

This experiment is conducted on an emotion classification task based on the “Arousal–Valence” dimension. To address the severe class imbalance problem in the arousal and valence dimensions of the eSEE-d dataset, SMOTE is adopted to perform subject-independent oversampling on minority-class samples within the training set. This ensures that the contribution of each class of samples to model parameter updates tends to be balanced during training, preventing the model from overfitting due to sample bias. For experimental comparison, the baseline models consist of traditional machine learning algorithms, state-of-the-art methods proposed in other relevant studies, and other temporal models constructed in this work. Specifically, the machine learning category includes models such as SVM and Random Forest; the deep learning category covers models like CNN and DMLP; and the temporal model category comprises LSTM and TCN models. To ensure the fairness of comparison, the same input features and training parameters are adopted for all baseline models. After completing the benchmark test on the eSEE-d dataset, cross-session experiments are further conducted on the SEED-IV multimodal dataset to comprehensively evaluate the generalization ability of the proposed model.

#### 5.1.2. Experimental Environment

The experiment’s software and hardware configurations are as follows: the software uses Python 3.12.3 and relies on libraries including NumPy 2.0.2, Pandas 2.3.0, PyTorch 2.1.0, and Scikit-learn 1.3.2. The hardware includes an NVIDIA GeForce RTX 4060 GPU (From NVIDIA Corporation, Santa Clara, CA, USA) with 12 GB of video random access memory (VRAM).

#### 5.1.3. Experimental Details and Parameter Configuration

All models are trained with identical parameter inputs, and the input features are kept consistent across all models. The AdamW optimizer is adopted for training, combined with a cosine annealing learning rate scheduler. The detailed configuration and key parameters of TCFN are presented in Table 2.

#### 5.1.4. Dataset Preprocessing

The emotion classification task in this paper is based on the Arousal–Valence dual-dimensional framework introduced in Section 2. The model adopts the eSEE-d dataset as the development benchmark and is further validated on the multi-modal SEED-IV dataset.

##### eSEE-d Database

The eSEE-d is an ET dataset initially involving 56 participants, with 48 healthy subjects retained post-screening (27 females, 21 males, aged 18–47), yielding 476 valid samples. The experiment includes ten emotion videos covering five categories: Anger, Sadness, Disgust, Tenderness, and Neutral. Data are recorded at a sampling rate of 240 Hz, with a spatial accuracy of 0.60 deg and a spatial resolution of 0.02 deg, including gaze coordinates, pupil diameter (mm), and blink timing information. Emotion labels are mapped to Arousal and Valence levels based on Russell’s Circumplex Model of Affect, specifically: Anger/Disgust→High Arousal–Negative Valence (HANV), Sadness→Low Arousal–Negative Valence (LANV), Tenderness→Low Arousal–Positive Valence (LAPV), and Neutral→Medium Arousal–Medium Valence (MAMV). Temporal feature extraction is performed on the data according to the method mentioned earlier, and the preprocessing steps are as follows:

(1) Verify the duration of each experiment in the raw data and remove data with a missing duration exceeding half of the complete experiment to avoid the impact of erroneous data.
(2) Remove data with a confidence level lower than 95% and fill in the missing data via linear interpolation.
(3) Perform filtering on the data using median filtering.
(4) Extract 25 statistical eye movement features from the data, as shown in Table 3.
(5) One-way analysis of variance (ANOVA) is conducted on the statistical features, features with *p* < 0.05 are retained, and the final ANOVA results, as well as the selected features, are presented in Table 4 and Table 5.

After extracting temporal features, 80% of the raw data is randomly sampled as the training set in accordance with the data partitioning method of DMLP, and subject-independent oversampling is performed on the training set using SMOTE.

**Table 3:** Raw ET features extracted from the eSEE-d dataset.

Eye Movement Parameters	Extracted Features
Pupil diameter (mm)	Mean, Kurt, Skew, Var, CV
Fixation duration (ms)	Mean, Kurt, Skew, Var, CV
Saccade duration (ms)	Mean, Kurt, Skew, Var, CV
Saccade speed (°/s)	Mean, Kurt, Skew, Var, CV
Saccade distance	Mean, Kurt, Skew, Var, CV

Mean denotes the mean value, Var represents variance, CV stands for the coefficient of variation, Kurt indicates kurtosis, and Skew refers to skewness.

**Table 4:** Final selected ET Features for the eSEE-d dataset.

Eye Movement Parameters	Extracted Features
Pupil diameter(mm)	Mean, Var, CV
Fixation duration(ms)	Mean, Var, CV
Saccade duration(ms)	Kurt, Skew, CV
Saccade speed(°/s)	Kurt, Skew
Saccade distance	Kurt, Skew

**Table 5:** Results of one-way ANOVA for feature significance.

Feature	HA-MA	MA-LA	HA-LA	PV-MV	MV-NV	PV-NV
Pupil diameter Mean	**0.014**	0.112	0.357	**0.001**	0.061	0.521
Pupil diameter Var	0.065	0.124	**0.000**	0.068	0.314	**0.005**
Pupil diameter CV	0.681	**0.000**	**0.000**	**0.000**	0.689	**0.000**
Fixation duration Mean	**0.000**	**0.000**	0.821	**0.001**	0.072	**0.026**
Fixation duration Var	0.254	**0.001**	**0.023**	**0.000**	**0.000**	0.079
Fixation duration CV	**0.000**	**0.000**	0.115	**0.000**	**0.000**	0.514
Saccade duration Kurt	**0.000**	**0.000**	0.891	**0.034**	**0.000**	**0.017**
Saccade duration Skew	**0.000**	**0.000**	0.653	**0.026**	**0.000**	**0.011**
Saccade duration CV	**0.002**	0.121	**0.050**	0.967	**0.001**	**0.001**
Saccade speed Kurt	**0.000**	**0.002**	0.458	0.154	**0.000**	**0.000**
Saccade speed Skew	**0.000**	**0.001**	0.328	0.124	**0.000**	**0.000**
Saccade distance Kurt	**0.031**	**0.002**	0.701	0.053	**0.025**	0.216
Saccade distance Kurt	**0.009**	**0.006**	0.708	0.099	**0.002**	0.137

Statistically significant differences (*p* < 0.05) are in bold.

##### SEED-IV Dataset

The SEED-IV dataset was collected from 15 subjects who viewed 24 film clips, with both EEG and ET data recorded. Each clip had a duration of approximately two minutes and corresponded to four emotion categories: Neutral, Sadness, Fear, and Joy. Since this paper adopts the Arousal–Valence Model for emotion classification, these four emotions were mapped to the Valence–Arousal dimensions: Neutral→Medium Arousal–Medium Valence (MAMV), Sadness→Low Arousal–Negative Valence (LANV), Fear→High Arousal–Negative Valence (HANV), and Joy→High Arousal–Positive Valence (HAPV). The ET features adopted in this paper are consistent with those reported in the original study [11]. For data partitioning, two sessions were used as the training set, and the remaining one as the validation set. This 3-fold cross-validation strategy was performed to validate the model’s classification accuracy.

### 5.2. Experimental Results and Comparison

As established earlier, the emotion classification task is based on the Arousal–Valence dual-dimensional model, utilizing the previously defined ET feature extraction methods. In the experiments, Valence is categorized into three states: Negative, Neutral, and Positive. Arousal is divided into three levels: High, Medium, and Low. Specifically, the Arousal levels of Low, Medium, and High are labeled as LA, MA, and HA classes, respectively. Similarly, the Valence levels of Negative, Neutral, and Positive are denoted as NV, MV, and PV classes, respectively.

In the eSEE-d dataset, this paper classifies the data separately according to the arousal and valence dimensions, and 5-fold cross-validation is conducted in the experiments. The comparison results between the experimental outcomes and existing methods are presented in Table 6.

In the experiments, we compare TCFN with machine learning approaches and the DMLP method [15]. Additionally, we refer to the LSTM and TCN architectures proposed by Safari and Elmadjian [30,31] to construct the LSTM and TCN for the comparative experiments. The results demonstrate the superior performance of our proposed TCFN. Compared with SVM, the accuracy of TCFN is improved by 18.59% and 19.77%. This demonstrates the effectiveness of our temporal scheme and spatial feature scheme. Compared with the DMLP model, the accuracy is improved by 11.27% and 5.94%, respectively. This is because the DMLP only performs classification on full-time features while ignoring the temporal and spatial correlations of ET features. In addition, the accuracy of TCFN is more than 3% higher than that of the LSTM and TCN temporal models, which demonstrates that the spatial information of ET features extracted by our capsule features contributes to the final classification performance. Moreover, the results of the standard deviation indicate that the overall accuracy of TCFN has only minor fluctuations, verifying the stability of the model.

**Figure 3:**
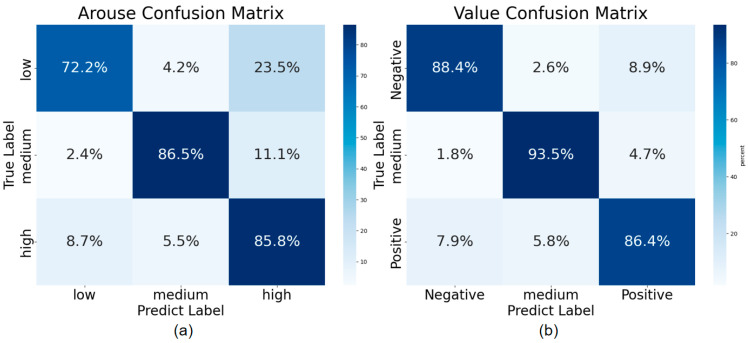
Experimental confusion matrix of the eSEE-d dataset. (**a**) represents the confusion matrix for the three classifications of arousal degree, and (**b**) represents the confusion matrix for the valence classification.

**Figure 4:**
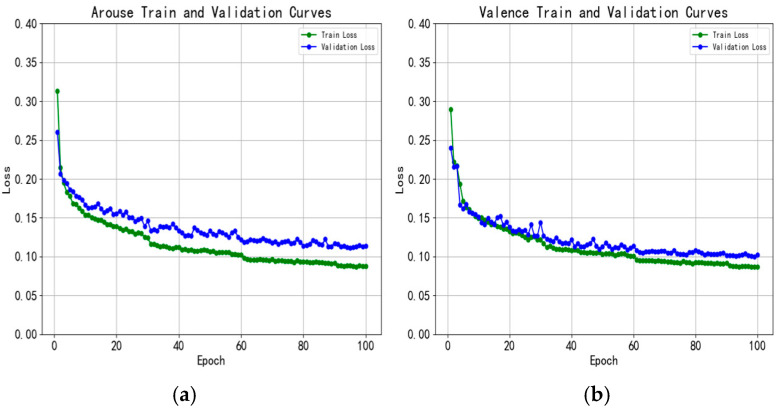
Training and testing loss curves on the eSEE-d dataset. (**a**) Denotes the loss curve for arousal three-class classification; (**b**) denotes the loss curve for valence three-class classification.

To further analyze the performance of our TCFN on the eSEE-d dataset, we present the normalized confusion matrix aggregated over the 5-fold cross-validation in Figure 3, and the training and testing loss curves are illustrated in Figure 4. The confusion matrices clearly show that the model’s recognition accuracy for Valence is higher than that for Arousal, this may be attributed to the fact that ET signals exhibit greater variability in terms of the valence dimension. Furthermore, in both Arousal and Valence classifications, the model achieves the best performance for the MA and MV categories, with accuracy exceeding 86% and 93%, respectively. For Arousal, the main confusion arises when 23.5% of LA samples are incorrectly classified as HA. For Valence, 8.9% of NV samples are misclassified as Positive Valence (PV). This confirms that ET parameters exhibit greater differentiation between the Medium/Neutral categories and other categories. This result is highly consistent with prior studies, demonstrating that our proposed TCFN can effectively learn accurate class boundaries.

**Figure 5:**
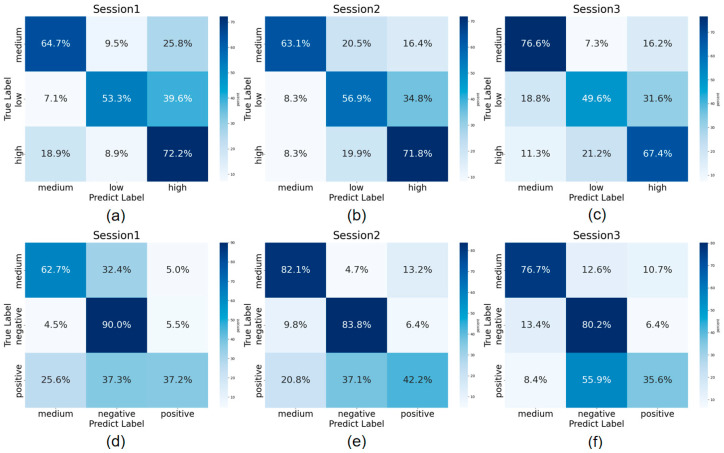
Confusion matrices for three cross-session experiments. (**a**–**c**) represent the confusion matrices for Arousal classification across the three cross-session experiments, and (**d**–**f**) represent the confusion matrices for Valence classification across the three cross-session experiments.

For the SEED-IV dataset, emotions were first mapped to the Arousal–Valence model for three-category classification (Arousal: LA/MA/HA; Valence: NV/MV/PV). The confusion matrices from the cross-session experiments are shown in Figure 5d–f correspond to the three Valence classification experiments. Across the three experiments, the accuracies for the MV category were 82.1%, 62.7%, and 76.7%, respectively, indicating significant differentiation from the other two categories. However, the test accuracy for PV and NV varied, suggesting these two categories were more difficult to distinguish. Figure 5a–c corresponds to the Arousal three-category classification. Based on the confusion matrices, the main error source across the three Arousal experiments was that 30% of LA samples were incorrectly classified as HA, and nearly 20% of HA samples were misclassified as LA. Consistent with the Valence results, the MA category showed greater differentiation from the other two levels, with more evenly distributed errors. Thus, the results in Figure 5 confirm the conclusions from the eSEE-d dataset experiments, verifying the effectiveness of our model.

To compare our approach with other state-of-the-art methods and machine learning algorithms, this paper also conducted a four-category emotion classification task on the SEED-IV dataset using the original emotion labels. The classification results of various models are presented in the comparison Table 7. Because SEED-IV is a multimodal dataset combining EEG and ET signals, in addition to the models used earlier in the comparison of experimental results, EEG classification models are also included for comparative analysis. Compared to EmotionMeter—which achieved 72.39% classification accuracy using multimodal data—TCFN attained an average accuracy of 63.85% using only ET parameters in cross-session classification experiments. This is comparable to EmotionMeter’s 67% accuracy when using only ET parameters for within-session classification, demonstrating that our model also performs well in the four-category classification task on the SEED-IV dataset. Compared to machine learning methods, our TCFN achieves an accuracy improvement of at least 10%. In comparison with the LSTM and TCN models, the TCFN model improves the classification accuracy by at least 5%, and its standard deviation is lower than that of the TCN model, indicating that the TCFN model has stability. In comparative experiments with EEG-based classification models, methods such as aDAPE achieved accuracies of 52.76%, 62.29%, and 62.13% using EEG data from the SEED-IV dataset for emotion classification. Since methods like aDAPE adopt a Leave-One-Subject-Out (LOSO) data partitioning strategy and target different physiological parameters, the comparison results primarily serve to validate the effectiveness of our proposed method. However, comparing the results of the two datasets shows that although TCFN yields a certain improvement in accuracy in both cases, there remains considerable room for optimization regarding its cross-dataset performance.

### 5.3. Ablation Experiment

To verify the functional contribution of each module in TCFN, this section conducts ablation experiments on the capsule network module, dual-loss module, and MLP module. To align the ablation results more closely with the model’s performance in practical applications, the ablation dataset is used for three-classification training based on the SEED-IV cross-session arousal experiment, with 3-fold cross-validation implemented. The results of the ablation experiments are summarized in Table 8. The results show that removing the dual-loss module causes a 3.38% decrease in model accuracy, which demonstrates that the margin loss in the capsule network tends to induce classification errors in emotion classification tasks due to the ambiguous boundaries of such tasks, and the introduction of weighted cross-entropy loss can better optimize the classification outcomes. Moreover, after removing the capsule network, the model accuracy drops by 2.36% with the largest standard deviation, verifying that the spatial features extracted by the capsule network provide effective support for emotion classification and can further enhance the stability of the model. In addition, in the experiment where the MLP module is removed, the model accuracy decreases by 2.17%, indicating that the MLP can better capture the spatial information extracted by the capsule network and achieve more accurate classification results than when directly performing classification with the capsule network alone.

According to the results of average inference time per sample in Table 8, the TCFN incurs substantial computational overhead in the capsule network module, whereas the dual-loss module and MLP module have relatively low computational overhead. This is mainly because the capsule network adopts a dynamic routing mechanism, which requires operations such as high-dimensional vector multiplication in each iteration, thus becoming the primary computational bottleneck in this study.

## 6. Discussion

Experimental results demonstrate that the TCFN has advantages in subject-dependent ET-only emotion recognition tasks. Furthermore, ablation experiments verify the functions of different modules. In this section, the advantages and disadvantages of this method in ET-only emotion recognition tasks are discussed.

Emotional states are closely related to the manifestations of ET signals and also to the spatial correlations among different ET features. Therefore, the framework proposed in this paper uses capsule networks to extract spatial information, which can improve the classification accuracy of ET-only emotion recognition tasks and achieve better recognition performance. By utilizing MLP classification and dual-loss optimization, the TCFN closely links the spatial correlations among features captured by capsule networks with emotional categories, enhancing the effectiveness of feature propagation and effectively alleviating the information loss problem existing in capsule networks. In addition, dual-loss optimization improves the discriminative ability for complex samples, enabling the model to more accurately capture feature differences in complex samples and reduce the impact of margin loss on boundary ambiguity problems.

However, the TCFN has certain limitations. Although it achieved good results in subject-dependent experiments, its accuracy remains low in cross-session experiments using multimodal methods. This may be attributed to the fact that ET patterns vary under different stimuli, along with the inconsistency of individuals’ subjective perception of emotional states. This indicates that TCFN still has deficiencies in addressing individual differences. In addition, compared with other approaches, the capsule network adopted in this study incurs higher computational overhead, resulting in slower model inference.

## 7. Conclusions

This study focuses on the application bottlenecks of ET features in affective computing. To address three core issues—insufficient exploitation of ET features in multimodal fusion, inadequate specificity of the correlation between ET and emotions, and generalization limitations caused by individual differences—TCFN is proposed, which integrates a window feature module, a capsule network module, and an MLP classification module. A dual-loss joint optimization mechanism is adopted to achieve synergistic improvement of feature extraction and classification accuracy. Experimental results show that the proposed network yields performance improvements over existing deep learning methods in the three-classification tasks of arousal (83.27%) and valence (89.94%) on the eSEE-d dataset. Furthermore, it achieves an accuracy of 63.85% in the four-class emotion classification task on the SEED-IV dataset, outperforming baseline models and delivering competitive results among ET-only methods.

This study proposes an ET feature emotion classification network with temporal information and feature spatial dimensions. TCFN provides a solution for real-time and practical emotion classification using ET features. However, the model has certain limitations, such as high computational overhead and the impact of individual differences on the model. In future work, we will focus on cross-individual research and model computational efficiency and combine other non-invasive and easily accessible modalities to further propose an emotion classification architecture with better practical value.

## Figures and Tables

**Table 1:** Summary of research methods and features in ET-only emotion classification studies.

Researcher	Classification Model	Feature
Skaramagkas [14]	SVM, KNN	Statistical feature
Li [29]	DGCNN	Image features
Tarnowski [28]	SVM, LDA, KNN	Statistical feature
Skaramagkas [15]	DMLP	Statistical feature
Ours	TCFN	Temporal and spatial feature

**Table 2:** Parameter Configuration Table.

Category/Parameter	Value
Training and Optimization	
Optimizer	AdamW
Learning rate	1 × 10^−3^
Batch size	32
Epochs	100(eSEE-d), 20(SEED-IV)
Weight decay	1 × 10^−4^
Loss weight λ	0.1
Model Architecture	
PrimaryCaps	capsules_num = 16, capsule_dim = 8
DigitCaps	capsules_num = 3, capsule_dim = 8
Hidden layer1	128
Hidden layer2	256
Regularization	
Dropout	0.5

**Table 6:** Classification accuracy comparison of various methods on the eSEE-d dataset.

Method	Arouse (%) ± Std	Value (%) ± Std
TCFN	83.27 ± 0.84	89.94 ± 1.08
DMLP [15]	72 ± \	84 ± \
LSTM [30]	73.39 ± 1.55	86.00 ± 1.03
TCN [31]	80.76 ± 1.73	86.86 ± 0.96
SVM	64.68 ± 1.50	70.17 ± 1.03
RF	62.97 ± 1.33	56.50 ± 0.81

**Table 7:** Classification accuracy comparison of various methods on the SEED-IV dataset.

Method	SEED-IV (%) ± Std
TCFN	63.85 ± 1.79
EmotionMeter [11]	67.82 ± 18.04
TCN [31]	58.12 ± 2.62
LSTM [30]	52.81 ± 0.17
SVM	48 ± \
aDAPE [37]	52.76 ± 2.92
HMNN [38]	62.29 ± \
GMSS [39]	62.13 ± 8.33

**Table 8:** Results of ablation experiments.

Method	Accuracy (%) ± Std	Avg_inference_time (ms) ± Std
TCFN	74.67 ± 3.06	3.95 ± 1.42
w/o Capsule	72.31 ± 6.44	0.88 ± 1.04
w/o Loss	71.29 ± 3.37	2.54 ± 1.31
w/o MLP	72.50 ± 3.61	2.56 ± 1.36

## Data Availability

The database used in this study is publicly available at the following websites: eSEE-d—DOI:10.5281/zenodo.5775674/ (accessed on 6 November 2023); SEED-IV—https://bcmi.sjtu.edu.cn/home/seed/ (accessed on 11 March 2025).

## Abbreviations

The following abbreviations are used in this manuscript:

ET	Eye Tracking
MLP	Multi-Layer Perceptron
LA	Low Arousal
MA	Medium Arousal
HA	High Arousal
NV	Negative Valence
MV	Medium Valence
PV	Positive Valence
TCFN	Temporal Capsule Feature Network
LOSO	Leave-One-Subject-Out
SVM	Support Vector Machine
CNN	Convolutional Neural Network
RF	Random Forest
KNN	k-Nearest Neighbors
DMLP	Deep Multi-layer Perceptron
LR	Logistic Regression
NB	Naive Bayes
